# An Exact Model-Based Method for Near-Field Sources Localization with Bistatic MIMO System

**DOI:** 10.3390/s17040723

**Published:** 2017-03-30

**Authors:** Parth Raj Singh, Yide Wang, Pascal Chargé

**Affiliations:** Institute of Electronics and Telecommunications of Rennes (IETR), UMR CNRS 6164, Polytech Nantes, Rue Christian Pauc, BP 50609, 44306 Nantes CEDEX 3, France; parth-raj.singh@etu.univ-nantes.fr (P.R.S.); pascal.charge@univ-nantes.fr (P.C.)

**Keywords:** near-field sources localization, bistatic MIMO system, PARAFAC

## Abstract

In this paper, we propose an exact model-based method for near-field sources localization with a bistatic multiple input, multiple output (MIMO) radar system, and compare it with an approximated model-based method. The aim of this paper is to propose an efficient way to use the exact model of the received signals of near-field sources in order to eliminate the systematic error introduced by the use of approximated model in most existing near-field sources localization techniques. The proposed method uses parallel factor (PARAFAC) decomposition to deal with the exact model. Thanks to the exact model, the proposed method has better precision and resolution than the compared approximated model-based method. The simulation results show the performance of the proposed method.

## 1. Introduction

Sources localization has been an important field of research for several decades. It is widely used in radar, underwater sources localization, acoustics, medicine, robotics, etc. The sources can be classified as near and far fields. Because of the wide range of applications, most of the research works [1,2,3,4,5] are dedicated to far-field sources localization. However, near-field sources localization has some important applications, like airport security control, ground penetration radar, phonocardiography, and many more.

Most of the existing near-field sources localization techniques [6,7,8,9,10,11,12,13,14,15,16] are based on an approximated model. In practice, a near-field point source has a spherical wavefront [6], which implies a nonlinear model. The wavefront of a near-field source is usually approximated as quadric (quadratic surface) to reduce the complexity of the model [6,9]. However, the use of this approximation results in a systematic error, which inevitably deteriorates the accuracy of the estimation. The systematic error is like an offset added to the actual source position, which increases when the target gets close to the antenna array [6].

In recent years, multiple input, multiple output (MIMO) radar has drawn a lot of attention. The advantages and limitations of MIMO radar have been well summarized in [2,3]. Based on the placement and configuration of the antennas, MIMO radar systems can broadly be classified as distributed or colocated. MIMO radar with colocated antennas can further be classified as monostatic, bistatic, and multistatic. In a bistatic MIMO radar system, the transmitting and receiving arrays are separated by a large distance, but the antennas in each array are kept close to each other (colocated) as compared to the distances between targets and the arrays. When the same array is used as transmitter and receiver, the system is monostatic. The directions of arrival and departure are different in the case of a bistatic MIMO system, but are equal for a monostatic MIMO radar system. If the distance between the transmitting and receiving arrays of a bistatic MIMO system is negligible compared to the range of targets, it can be considered as a pseudo-monostatic MIMO system. The work by Guo et al. [10] provides a subspace-based near-field sources localization method for a pseudo-monostatic MIMO radar system. A near-field sources localization method based on an approximated model with a bistatic MIMO system was proposed in [15]. Recently, in [17], a method based on the exact model of the received signal has been proposed to locate near-field targets using a bistatic MIMO system composed of linearly-aligned transmitting and receiving arrays. This paper is an improvement and an extension of the method in [17]. The major differences between the two are:

1. The method in [17] is specific to the linearly-aligned transmitting and receiving arrays, whereas this paper deals with any configuration for the transmitting and receiving uniform linear arrays (ULAs) (3D situation).
2. Due to the linearly-aligned transmitting and receiving arrays, the cost function in [17] has only two variables. However, the generalized 3D configuration in this paper results in a three-variable cost function which is much more difficult to deal with. Thus, in this paper, we propose a better and more efficient approach based on an overdetermined system of linear equations.

In [15], four parameters—namely, the angle of arrival, the angle of departure of a target, and the distances (ranges) from the target to the transmitting and receiving arrays—are used to localize the target, but there are some redundancies because three coordinates are sufficient to define the position of a target. Therefore, in this paper, we use Cartesian coordinates to formulate the signal model and express the localization error.

There are many existing methods to localize sources from an array of sensors, such as Capon’s method, multiple signal classification (MUSIC), estimation of signal parameter via rotational invariance techniques (ESPRIT), propagator method, and tensor decomposition method [1,5]. Among the methods listed above, the tensor decomposition method directly estimates the whole directional matrix instead of the directional parameters [18], which facilitates the estimation of the directional parameters from a nonlinear model such as the exact model in near-field situation. Consequently, the proposed exact model-based method uses the tensor decomposition. Tensor-based models and techniques are well adapted to MIMO radar because tensors allow coping with large systems (three or more dimensions). The received signal in the case of a bistatic MIMO system can be organized as a three-way tensor. Three-way tensors have attracted a lot of attention because they are the simplest form of tensor after a matrix and can be decomposed into unique factors, contrary to a matrix. Kruskal [19] provides a detailed study of the rank and uniqueness in the decomposition of a three-way tensor. The tensor decomposition has already been used for multiple far-field sources localization with bistatic MIMO radar [5].

There exist many tensor decomposition techniques, such as Tucker, parallel factor analysis (PARAFAC), and block component decomposition [20]. PARAFAC is often used in array signal processing thanks to its uniqueness in the decomposition of tensors under some mild conditions [18]. Thus, in this paper, we select PARAFAC to decompose the three-way tensor of the received signal to obtain the directional matrices of arrival and departure. From the existing work on the application of PARAFAC to the localization of targets, we can observe that it is mainly proposed for far-field target localization. In this paper, we extend it to the near-field situation. Once the directional matrices are estimated, an optimization method can be used to obtain the directional parameters.

To summarize, this paper focuses on the use of an exact model of the received signals of near-field sources to get better performance than the existing approximated model-based techniques for near-field sources localization with a bistatic MIMO system. Due to the nonlinear nature of the exact model, the PARAFAC decomposition is used, and an optimization technique is developed to efficiently solve this problem.

The remainder of the paper is organized as follows. In Section 2, a detailed signal model is constructed for a bistatic MIMO radar system based on the spherical wavefront of an incoming wave by taking the exact propagation model in the near-field situation into account. Section 3 provides a short presentation of the method proposed in [15]. In Section 4, the proposed method is described. Finally, some simulation results are presented to compare the performance of the proposed method with the method presented in [15], followed by some discussion and conclusions.

### Notations

In the following, a bold lower case character (e.g., a) represents a vector, whereas a bold upper case character (e.g., A) denotes a matrix. A tensor is denoted by a bold upper case calligraphic font (e.g., Y). ${\left[•\right]}^{T}$, ${\left[•\right]}^{+}$, and ${∥•∥}_{F}$ represent, respectively, the transpose, left pseudo-inverse, and Frobenius norm of a matrix or vector. ⊡ is the Khatri–Rao (column-wise Kronecker) product operator. The cardinal number of a set is represented by c(•). ∠(•) stands for the principal value of the angle (or argument) of a complex number. D{a} represents the diagonal matrix with all the components of vector a as its diagonal elements. E{•} is the expected value.

## 2. Signal Model

Let *P* be the number of narrow-band stationary point sources in the near-field region of a bistatic MIMO system with ULAs. In the following, *M* and *N* represent, respectively, the number of antennas in the transmitting and receiving arrays of the bistatic MIMO system.

For such a bistatic MIMO system, the *L* samples of the received matched signal in the presence of *P* stationary point sources can be written as [4]

(1) $$\begin{array}{cc} Y_{M} & =\left(A_{e}⊡A_{r}\right)S^{T}+W_{M} \end{array}$$

where $A_{e}\in C^{M\times P}$ and $A_{r}\in C^{N\times P}$ contain the directional vectors of departure and arrival, respectively, $S\in C^{L\times P}$ is the matrix of the complex-valued reflection coefficients of targets, and $W_{M}\in C^{MN\times L}$ is an additive noise matrix composed of spatially- and temporally-independent elements, and each element is a zero mean Gaussian random variable with variance $\sigma^{2}$. The reflection coefficients are assumed to be different for each target and randomly changing with each sample. In other words, we consider a Swerling model II case, which makes S a full rank matrix [21]. The *p*th columns of $A_{e}$ and $A_{r}$—denoted by $a_{e_{p}}$ and $a_{r_{p}}$, respectively—are given by

(2) $$\begin{array}{c} a_{e_{p}}={\left[a_{e_{\left(1-m_{o},\;p\right)}},\cdots ,1,\cdots ,a_{e_{\left(M-m_{o},\;p\right)}}\right]}^{T} \end{array}$$

and

(3) $$\begin{array}{c} a_{r_{p}}={\left[a_{r_{\left(p,\;1-n_{o}\right)}},\cdots ,1,\cdots ,a_{r_{\left(p,\;N-n_{o}\right)}}\right]}^{T} \end{array}$$

where $m_{o}$ and $n_{o}$ are the indexes of the reference elements of the transmitting and receiving arrays, respectively, $a_{e_{\left(m,\;p\right)}}=e^{-j\;2\;\pi \;\delta_{e_{\left(m,\;p\right)}}/\lambda }$, and $a_{r_{\left(p,\;n\right)}}=e^{-j\;2\;\pi \;\delta_{r_{\left(p,\;n\right)}}/\lambda }$. $m\in \{1-m_{o},\;2-m_{o},\cdots ,M-m_{o}\}$ and $n\in \{1-n_{o},\;2-n_{o},\cdots ,\;N-n_{o}\}$ are the relative indexes of the respective arrays. λ is the wavelength of the carrier. $\delta_{e_{\left(m,\;p\right)}}$ is the difference between the distance traveled by the transmitted signal from the *m*th transmitting antenna to the *p*th target and the distance traveled by the transmitted signal from the 0th transmitting antenna to the *p*th target, which can be expressed as

(4) $$\begin{array}{c} \delta_{e_{\left(m,\;p\right)}}=\sqrt{\rho_{e_{p}}^{2}+m^{2}\;d_{e}^{2}-2\;m\;d_{e}\;\rho_{e_{p}}\cos\left(\theta_{e_{p}}\right)}-\rho_{e_{p}} \end{array}$$

where $\rho_{e_{p}}$ and $\theta_{e_{p}}$ are respectively the range and angle of departure of the *p*th target with respect to the reference transmitting antenna indexed by $m_{o}$, and $d_{e}$ is the inter-element spacing in the transmitting array. Similarly, $\delta_{r_{\left(p,\;n\right)}}$ is the difference between the distance traveled by the reflected signal from the *p*th target to the *n*th receiving antenna and the distance traveled by the reflected signal from the *p*th target to the 0th receiving antenna, which can be expressed as

(5) $$\begin{array}{c} \delta_{r_{\left(p,\;n\right)}}=\sqrt{\rho_{r_{p}}^{2}+n^{2}\;d_{r}^{2}-2\;n\;d_{r}\;\rho_{r_{p}}\cos\left(\theta_{r_{p}}\right)}-\rho_{r_{p}} \end{array}$$

where $\rho_{r_{p}}$ and $\theta_{r_{p}}$ are, respectively, the range and angle of arrival of the *p*th target with respect to the reference receiving antenna indexed by $n_{o}$ and $d_{r}$ is the inter-element spacing in the receiving array [6].

$Y_{M}$ in Equation (1) can be considered as a block matrix, $Y_{M}={\left[{\overset{˘}{Y}}_{1-m_{o}}^{T},\;{\overset{˘}{Y}}_{2-m_{o}}^{T},\;\cdots ,\;{\overset{˘}{Y}}_{M-m_{o}}^{T}\right]}^{T}$. The *m*th sub-matrix of $Y_{M}$ (i.e., ${\overset{˘}{Y}}_{m}\in C^{N\times L}$) can be expressed as

(6) $${\overset{˘}{Y}}_{m}=A_{r}D_{m}S^{T}+{\overset{˘}{W}}_{m}$$

where $D_{m}=D\left\{\left[a_{e_{\left(m,\;1\right)}},\;a_{e_{\left(m,\;2\right)}},\;\cdots ,\;a_{e_{\left(m,\;P\right)}}\right]\right\}$ and ${\overset{˘}{W}}_{m}$ is the corresponding noise sub-matrix.

## 3. Approximated Model-Based Method Proposed in [15]

Most of the existing near-field sources localization techniques [6,7,8,9,11,14] use an approximated model, and ULA is often used in the approximated model-based methods. The approximated path differences—which are the second-order Taylor approximations of Equations (4) and (5)—can respectively be written as [6]

(7) $${\tilde{\delta }}_{e_{\left(m,\;p\right)}}=-m\;d_{e}\;\cos\left(\theta_{e_{p}}\right)+\frac{m^{2}\;d_{e}^{2}}{2\;\rho_{e_{p}}}\;\sin^{2}\left(\theta_{e_{p}}\right)$$

and

(8) $${\tilde{\delta }}_{r_{\left(p,\;n\right)}}=-n\;d_{r}\;\cos\left(\theta_{r_{p}}\right)+\frac{n^{2}\;d_{r}^{2}}{2\;\rho_{r_{p}}}\;\sin^{2}\left(\theta_{r_{p}}\right).$$

In [15], a subspace-based method is used to estimate four parameters: two ranges ($\rho_{e_{p}}$ and $\rho_{r_{p}}$) and two directional angles ($\theta_{e_{p}}$ and $\theta_{r_{p}}$) of a near-field target by using a bistatic MIMO system with inter-element spacing of λ/4 in each ULA. M∈{2μ+1:μ∈N,μ>1}, $N\in \{2\;\overset{˘}{\mu }+1:\overset{˘}{\mu }\in N\}$, $m_{o}=\left(M+1\right)/2$, $n_{o}=\left(N+1\right)/2$, $d_{e}\leq \lambda /4$, and $d_{r}\leq \lambda /4$ are the necessary conditions of [15].

In an approximated model-based method like [15], $A_{e}$ and $A_{r}$ are assumed to be constructed by ${\tilde{\delta }}_{e_{\left(m,\;p\right)}}$ and ${\tilde{\delta }}_{r_{\left(p,\;n\right)}}$, respectively. Therefore, in this case, $D_{m}$ in Equation (6) can be expressed as

(9) $$D_{m}=D\left\{\left[e^{j\left(m\;\omega_{e_{1}}-m^{2}\;\phi_{e_{1}}\right)},\;e^{j\left(m\;\omega_{e_{2}}-m^{2}\;\phi_{e_{2}}\right)},\;\cdots ,\;e^{j\left(m\;\omega_{e_{P}}-m^{2}\;\phi_{e_{P}}\right)}\right]\right\}$$

where $\omega_{e_{p}}=2\;\pi \;d_{e}\cos\left(\theta_{e_{p}}\right)/\lambda$ and $\phi_{e_{p}}=\pi \;d_{e}^{2}\sin^{2}\left(\theta_{e_{p}}\right)/\left(\lambda \;\rho_{e_{p}}\right)$. In [15], four cross-covariance matrices between ${\overset{˘}{Y}}_{m}$ for m∈{−2,−1,1,2} and ${\overset{˘}{Y}}_{0}$ are constructed. The eigenvalues of $R_{-2}\;R_{-1}^{+}$ and $R_{2}\;R_{1}^{+}$ are used to get $\rho_{e_{p}}$ and $\theta_{e_{p}}$, and their eigenvectors are used to obtain $\rho_{r_{p}}$ and $\theta_{r_{p}}$, where $R_{m}=E\left\{{\overset{˘}{Y}}_{m}\;{\overset{˘}{Y}}_{0}^{H}\right\}$. More details can be found in [15].

## 4. Proposed Exact Model-Based Position Estimation Method

**Figure 1:**
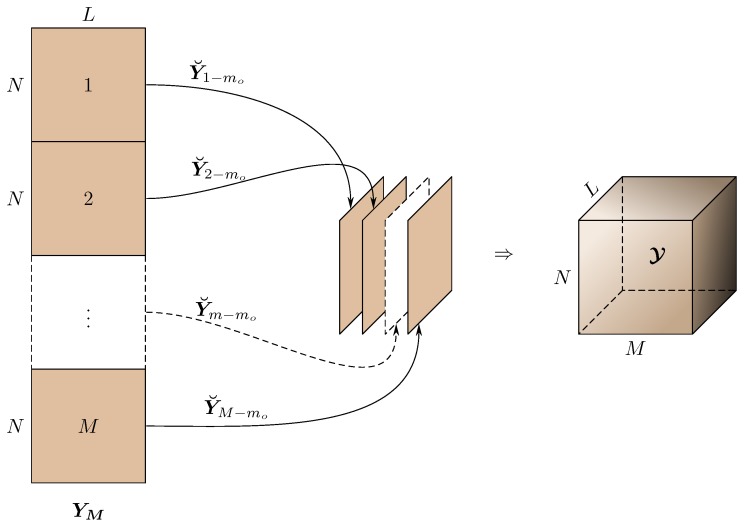
Tensorization.

Every element of $Y_{M}$ in Equation (1) is associated with three parameters related, respectively, to the receiving antenna, transmitting antenna, and time sample. Therefore, $Y_{M}$ can be rearranged like a three-way tensor $Y\in C^{N\times M\times L}$, as shown in Figure 1. Creating a tensor out of lower dimensional data is known as tensorization [20].

PARAFAC decomposition of tensor Y is used to get the estimates of $A_{r}$, $A_{e}$, and S matrices [5]. Tensor operations are usually performed in its equivalent matrix form [5,22,23]. The process of creating a matrix out of a tensor is known as matricization [20]. Like $Y_{M}$, Y can be matricized into the following two additional matrices

(10) $$Y_{L}=\left(S⊡A_{e}\right){A_{r}}^{T}+W_{L}$$

and

(11) $$Y_{N}=\left(A_{r}⊡S\right){A_{e}}^{T}+W_{N}.$$

According to the least squares principle, the following objective functions can be written from Equations (1), (10) and (11)

(12) $$\hat{S}=\underset{S}{arg min}\left\{{∥Y_{M}-\left(A_{e}⊡A_{r}\right)S^{T}∥}_{F}^{2}\right\},$$

(13) $${\hat{A}}_{r}=\underset{A_{r}}{arg min}\left\{{∥Y_{L}-\left(S⊡A_{e}\right){A_{r}}^{T}∥}_{F}^{2}\right\},$$

and

(14) $${\hat{A}}_{e}=\underset{A_{e}}{arg min}\left\{{∥Y_{N}-\left(A_{r}⊡S\right){A_{e}}^{T}∥}_{F}^{2}\right\}$$

where ${\hat{A}}_{r}$, ${\hat{A}}_{e}$, and $\hat{S}$ denote the estimated values of $A_{r}$, $A_{e}$, and S respectively.

The trilinear alternating least squares (TALS) algorithm is a classical method to minimize the above objective functions [5,22,23]. Least squares estimates of Equations (12)–(14) are given by

(15) $$\hat{S}={\left[{\left(A_{e}⊡A_{r}\right)}^{+}Y_{M}\right]}^{T},$$

(16) $${\hat{A}}_{r}={\left[{\left(S⊡A_{e}\right)}^{+}Y_{L}\right]}^{T},$$

and

(17) $${\hat{A}}_{e}={\left[{\left(A_{r}⊡S\right)}^{+}Y_{N}\right]}^{T}.$$

In the TALS algorithm, Equations (15)–(17) are alternatively updated with the new values of ${\hat{A}}_{r}$, ${\hat{A}}_{e}$, and $\hat{S}$ until a stopping criteria is met. $∥Y_{M}-{\left(A_{e}⊡A_{r}\right)S^{T}∥}_{F}^{2}<\epsilon_{\mathrm{tol}}$ is often used as the stopping condition, where $\epsilon_{\mathrm{tol}}$ is the tolerance. In practice, the algorithm given in [24] is used for PARAFAC decomposition, which uses compression, line search, normalization, etc. to accelerate the TALS method.

According to [19], the matrices obtained by PARAFAC decomposition of a three-way tensor are scaled and permuted. The permutation has no impact because the matrices’ columns are paired. However, in the proposed method, the scaling factor must be removed by dividing all the elements of the directional vectors with their corresponding reference elements.

**Figure 2:**
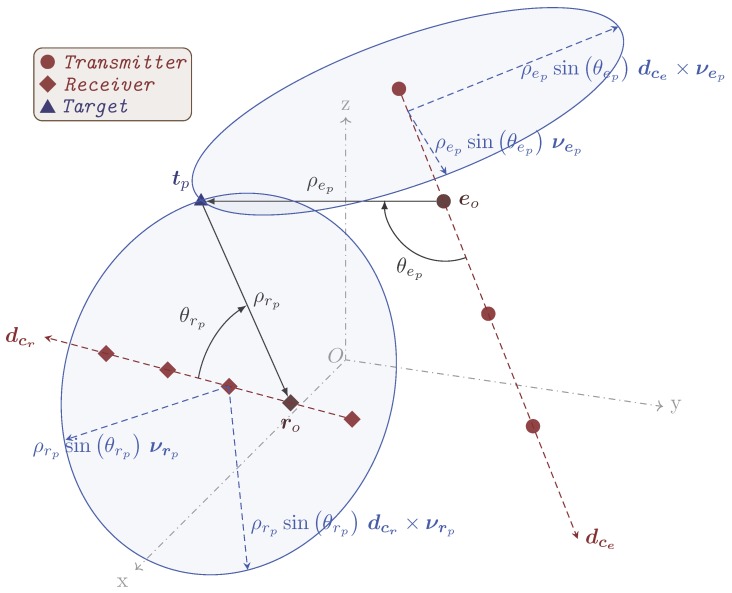
General configuration of a bistatic multiple input, multiple output (MIMO) radar system with linear arrays.

To define the Cartesian coordinates of a target, let us assume a general configuration of bistatic MIMO system as shown in Figure 2. In the case of a ULA, the unit vector along the array and the position vector of the reference antenna of the corresponding array are sufficient to obtain the position vectors of the remaining antennas of that array. In the figure, $e_{o}$ and $r_{o}$ are the position vectors of the reference transmitting and receiving antennas, respectively, with respect to the origin of the Cartesian coordinate system, and $d_{c_{e}}$ and $d_{c_{r}}$ are the unit vectors along the transmitting and receiving arrays, respectively. $t_{p}={\left[x_{t_{p}},\;y_{t_{p}},\;z_{t_{p}}\right]}^{T}$ represents the position vector of the *p*th target. In 3D space, the range and directional angle of a target with respect to a linear array make a circle related to the base of a cone with the range as its slant height and the directional angle as its half angle. In the bistatic case, we have two such circles (shown in Figure 2). The target is located at the intersection of these two circles. In the figure, $\nu_{e_{p}}$ and $\nu_{r_{p}}$ are unit vectors on the planes of the respective circles. The parametric equations of the circles can be written as

(18) $$\psi_{e_{p}}\left(\varphi_{e}\right)=\rho_{e_{p}}\;\sin\left(\theta_{e_{p}}\right)\;\left[\cos\left(\varphi_{e}\right)\;\nu_{e_{p}}+\sin\left(\varphi_{e}\right)\;d_{c_{e}}\times \nu_{e_{p}}\right]+\rho_{e_{p}}\;\cos\left(\theta_{e_{p}}\right)d_{c_{e}}+e_{o}$$

and

(19) $$\psi_{r_{p}}\left(\varphi_{r}\right)=\rho_{r_{p}}\;\sin\left(\theta_{r_{p}}\right)\;\left[\cos\left(\varphi_{r}\right)\;\nu_{r_{p}}+\sin\left(\varphi_{r}\right)\;d_{c_{r}}\times \nu_{r_{p}}\right]+\rho_{r_{p}}\;\cos\left(\theta_{r_{p}}\right)d_{c_{r}}+r_{o}$$

where × denotes the cross-product operation between two vectors; $\psi_{e_{p}}\left(\varphi_{e}\right)$ and $\psi_{r_{p}}\left(\varphi_{r}\right)$ are the position vectors of a point on the respective circles at $\varphi_{e}$ and $\varphi_{r}$, respectively. The equation parameters $\varphi_{e}$ and $\varphi_{r}$ independently vary from 0 to 2π rad to completely sweep the respective circles.

The ranges and directional angles can be expressed in terms of the Cartesian coordinates as $\rho_{e_{p}}\;=\;{∥t_{p}-e_{o}∥}_{F}$, $\rho_{r_{p}}={∥t_{p}-r_{o}∥}_{F}$, $\theta_{e_{p}}=\arccos\left[{\left(t_{p}-e_{o}\right)}^{T}d_{c_{e}}/\rho_{e_{p}}\right]$, and $\theta_{r_{p}}=\arccos\left[{\left(t_{p}-r_{o}\right)}^{T}d_{c_{r}}/\rho_{r_{p}}\right]$. Thus, according to Equations (2)–(5), $a_{e_{p}}$ and $a_{r_{p}}$ can respectively be determined by $t_{p}$ as

(20) $$a_{e_{\left(m,\;p\right)}}=e^{-j\;2\;\pi \left(\sqrt{{∥t_{p}-e_{o}∥}_{F}^{2}+m^{2}\;d_{e}^{2}-2\;m\;d_{e}{\left(t_{p}-e_{o}\right)}^{T}d_{c_{e}}}-{∥t_{p}-e_{o}∥}_{F}\right)/\lambda }$$

and

(21) $$a_{r_{\left(p,\;n\right)}}=e^{-j\;2\;\pi \left(\sqrt{{∥t_{p}-r_{o}∥}_{F}^{2}+n^{2}\;d_{r}^{2}-2\;n\;d_{r}{\left(t_{p}-r_{o}\right)}^{T}d_{c_{r}}}-{∥t_{p}-r_{o}∥}_{F}\right)/\lambda }.$$

Then, a direct approach to estimate $t_{p}$ could be the minimization of the following cost function

(22) $$\begin{array}{c} {\hat{t}}_{p}=\underset{t_{p}}{arg min}\left\{{∥{\hat{a}}_{e_{p}}/{\hat{a}}_{e_{\left(0,\;p\right)}}-a_{e_{p}}∥}_{F}^{2}+{∥{\hat{a}}_{r_{p}}/{\hat{a}}_{r_{\left(p,\;0\right)}}-a_{r_{p}}∥}_{F}^{2}\right\} \end{array}$$

where ${\hat{a}}_{e_{p}}$ and ${\hat{a}}_{r_{p}}$ are the estimated directional vectors obtained from the PARAFAC decomposition, and ${\hat{a}}_{e_{\left(0,\;p\right)}}$ and ${\hat{a}}_{r_{\left(p,\;0\right)}}$ are their respective reference elements used here to remove the scaling factor in the decomposition of the received signal tensor.

Even though a near-field region occupies a finite space, minimizing Equation (22) by using grid search or Newton’s method is computationally expensive. Therefore, we choose an indirect method in which we estimate the ranges and directional angles, followed by the estimation of the coordinates.

Rearranging Equations (4) and (5), we can obtain

(23) $$\begin{array}{cc} 2\;m\;d_{e}\;\rho_{e_{p}}\;\cos\left(\theta_{e_{p}}\right)+2\;{\hat{\delta }}_{e_{\left(m,\;p\right)}}\;\rho_{e_{p}} & =m^{2}\;d_{e}^{2}-{\hat{\delta }}_{e_{\left(m,\;p\right)}}^{2} \end{array}$$

(24) $$\begin{array}{cc} 2\;n\;d_{r}\;\rho_{r_{p}}\;\cos\left(\theta_{r_{p}}\right)+2\;{\hat{\delta }}_{r_{\left(p,\;n\right)}}\;\rho_{r_{p}} & =n^{2}\;d_{r}^{2}-{\hat{\delta }}_{r_{\left(p,\;n\right)}}^{2} \end{array}$$

where ${\hat{\delta }}_{e_{\left(m,\;p\right)}}$ and ${\hat{\delta }}_{r_{\left(p,\;n\right)}}$ are the estimated path differences which can be directly obtained from the estimated directional vectors as follows

(25) $${\hat{\delta }}_{e_{\left(m,\;p\right)}}=-\lambda \left[U\left\{∠\left({\hat{a}}_{e_{\left(m,\;p\right)}}\right)\right\}\right.-\left.U\left\{∠\left({\hat{a}}_{e_{\left(0,\;p\right)}}\right)\right\}\right]/2\pi$$

and

(26) $${\hat{\delta }}_{r_{\left(p,\;n\right)}}=-\lambda \left[U\left\{∠\left({\hat{a}}_{r_{\left(p,\;n\right)}}\right)\right\}\right.-\left.U\left\{∠\left({\hat{a}}_{r_{\left(p,\;0\right)}}\right)\right\}\right]/2\pi$$

where $U\left\{•\right\}$ represents the unwrapped value of the argument [25]. Equations (25) and (26) can be described as follows:

1. Get the directional vectors ${\hat{a}}_{e_{p}}$ and ${\hat{a}}_{r_{p}}$ from the PARAFAC decomposition.
2. Extract the arguments of all the components of ${\hat{a}}_{e_{p}}$ and ${\hat{a}}_{r_{p}}$.
3. Unwrap the phase vectors obtained from Step 2.
4. Subtract the unwrapped phase corresponding to ${\hat{a}}_{e_{\left(0,\;p\right)}}$ and ${\hat{a}}_{r_{\left(p,\;0\right)}}$ from all the components of the unwrapped phase vector of ${\hat{a}}_{e_{p}}$ and ${\hat{a}}_{r_{p}}$, respectively.
5. Divide each component of the normalized phase vectors obtained from the above step by −2π/λ to get ${\hat{\delta }}_{e_{\left(m,\;p\right)}}$ and ${\hat{\delta }}_{r_{\left(p,\;n\right)}}$.

In practice, M≥2 and N≥2; therefore, Equations (23) and (24) can be considered as an overdetermined system of linear equations in $\rho_{e_{p}}\;\cos\left(\theta_{e_{p}}\right)$ and $\rho_{e_{p}}$ and $\rho_{r_{p}}\;\cos\left(\theta_{r_{p}}\right)$ and $\rho_{r_{p}}$, respectively, which can be solved by the total least squares method [26]. Let ${\left[u_{1_{p}},u_{2_{p}},u_{3_{p}}\right]}^{T}$ be the right-singular-vector associated with the smallest singular value of the following matrix formed by the coefficients of Equation (23)

(27) $$\left[\begin{array}{ccc} 2\;\left(1-m_{o}\right)\;d_{e} & 2\;{\hat{\delta }}_{e_{\left(1-m_{o},\;p\right)}} & {\left(1-m_{o}\right)}^{2}\;d_{e}^{2}-{\hat{\delta }}_{e_{\left(1-m_{o},\;p\right)}}^{2}\\ 2\;\left(2-m_{o}\right)\;d_{e} & 2\;{\hat{\delta }}_{e_{\left(2-m_{o},\;p\right)}} & {\left(2-m_{o}\right)}^{2}\;d_{e}^{2}-{\hat{\delta }}_{e_{\left(2-m_{o},\;p\right)}}^{2}\\ \vdots & \vdots & \vdots \\ 2\;\left(M-m_{o}\right)\;d_{e} & 2\;{\hat{\delta }}_{e_{\left(M-m_{o},\;p\right)}} & {\left(M-m_{o}\right)}^{2}\;d_{e}^{2}-{\hat{\delta }}_{e_{\left(M-m_{o},\;p\right)}}^{2} \end{array}\right].$$

The estimated range and angle of departure can respectively be computed by ${\hat{\rho }}_{e_{p}}=-u_{2_{p}}/u_{3_{p}}$ and ${\hat{\theta }}_{e_{p}}=\arccos\left(u_{1_{p}}/u_{2_{p}}\right)$. Similarly, let ${\left[v_{1_{p}},\;v_{2_{p}},\;v_{3_{p}}\right]}^{T}$ be the right-singular-vector associated with the smallest singular value of the following matrix formed by the coefficients of Equation (24)

(28) $$\left[\begin{array}{ccc} 2\;\left(1-n_{o}\right)\;d_{r} & 2\;{\hat{\delta }}_{r_{\left(p,\;1-n_{o}\right)}} & {\left(1-n_{o}\right)}^{2}\;d_{r}^{2}-{\hat{\delta }}_{r_{\left(p,\;1-n_{o}\right)}}^{2}\\ 2\;\left(2-n_{o}\right)\;d_{r} & 2\;{\hat{\delta }}_{r_{\left(p,\;2-n_{o}\right)}} & {\left(2-n_{o}\right)}^{2}\;d_{r}^{2}-{\hat{\delta }}_{r_{\left(p,\;2-n_{o}\right)}}^{2}\\ \vdots & \vdots & \vdots \\ 2\;\left(N-n_{o}\right)\;d_{r} & 2\;{\hat{\delta }}_{r_{\left(p,\;N-n_{o}\right)}} & {\left(N-n_{o}\right)}^{2}\;d_{r}^{2}-{\hat{\delta }}_{r_{\left(p,\;N-n_{o}\right)}}^{2} \end{array}\right].$$

The estimated range and angle of arrival can respectively be computed by ${\hat{\rho }}_{r_{p}}=-v_{2_{p}}/v_{3_{p}}$ and ${\hat{\theta }}_{r_{p}}=\arccos\left(v_{1_{p}}/v_{2_{p}}\right)$.

The estimated ranges and directional angles can be used in Equations (18) and (19) to construct the parametric equations of the circles. As mentioned before, the required coordinates are at the intersection of these circles. However, due to the estimation error and noise, the circles may not intersect; thus, the following minimization problem can be solved:

(29) $$\begin{array}{c} \left({\hat{\varphi }}_{e},\;{\hat{\varphi }}_{r}\right)=\underset{\left(\varphi_{e},\;\varphi_{e}\right)}{arg min}\left\{{∥\psi_{e_{p}}\left(\varphi_{e}\right)-\psi_{r_{p}}\left(\varphi_{r}\right)∥}_{F}^{2}\right\}. \end{array}$$

A coarse solution of Equation (29) can be calculated by exhaustive grid search, and then it can be finely tuned by Newton’s method. Solving Equation (29) is less complex than solving Equation (22). Finally, the position vector of the *p*th target can be computed as ${\hat{t}}_{p}=\psi_{e_{p}}\left({\hat{\varphi }}_{e}\right)$, ${\hat{t}}_{p}=\psi_{r_{p}}\left({\hat{\varphi }}_{r}\right)$, or the average of these two position vectors.

Algorithm 1 provides a summary of the proposed method.

**Algorithm 1** Algorithm of the proposed method.
Construct the three-way tensor Y from the received data.Estimate $A_{e}$ and $A_{r}$ from Y using PARAFAC decomposition.Use Equations (25) and (26) to obtain ${\hat{\delta }}_{e_{\left(m,\;p\right)}}$ and ${\hat{\delta }}_{r_{\left(p,\;n\right)}}$ from the estimated $A_{e}$ and $A_{r}$, respectively.Create the system of linear equations by substituting ${\hat{\delta }}_{e_{\left(m,\;p\right)}}$ and ${\hat{\delta }}_{r_{\left(p,\;n\right)}}$ in Equations (23) and (24), respectively, for all the values of *m* and *n* for each target.Separately solve each system of linear equations created in step 4 using the total least squares technique to obtain ${\hat{\rho }}_{e_{p}}$, ${\hat{\theta }}_{e_{p}}$, ${\hat{\rho }}_{r_{p}}$, and ${\hat{\theta }}_{r_{p}}$.Substitute the four estimated location parameters in Equations (18) and (19) to obtain the parametric equations of the circles, and minimize (29) to estimate ${\hat{\varphi }}_{e}$ and ${\hat{\varphi }}_{r}$.Finally, substitute ${\hat{\varphi }}_{e}$ and ${\hat{\varphi }}_{r}$ along with ${\hat{\rho }}_{e_{p}}$, ${\hat{\theta }}_{e_{p}}$, ${\hat{\rho }}_{r_{p}}$, and ${\hat{\theta }}_{r_{p}}$ in Equations (18) and (19) to get the estimated coordinates ${\hat{t}}_{p}$ of the *p*th target.

## 5. Simulation Results

In the following simulations, the performance of the proposed method and the method in [15] is compared, with M=5, N=9, $m_{o}=3$, $n_{o}=5$, and $d_{e}=d_{r}=\lambda /4$ to satisfy the necessary requirements of [15]. Throughout the simulation, λ is used as the unit of length. The remaining MIMO system configuration parameters are $e_{o}={\left[0.5\lambda ,\;\lambda ,\;1.5\lambda \right]}^{T}$, $r_{o}={\left[0.4\lambda ,-0.3\lambda ,-0.2\lambda \right]}^{T}$, $d_{c_{e}}=\;{\left[0.3420,\;0.5000,\;-0.7956\right]}^{T}$, and $d_{c_{r}}={\left[0.8660,\;-0.1736,\;0.4689\right]}^{T}$, which are chosen randomly such that there exists a significant near-field region shared by both ULAs.

According to the three estimated coordinates, the root mean square error (RMSE) associated with the position estimation of the *p*th target is calculated as follows:

(30) $$\begin{array}{c} {\mathrm{RMSE}}_{p}=\sqrt{\frac{1}{K}\sum_{k=1}^{K}{∥{\hat{t}}_{p}\left(k\right)-t_{p}∥}_{F}^{2}} \end{array}$$

where *K* is the number of Monte Carlo iterations, ${\hat{t}}_{p}\left(k\right)$ represents the estimated position at the *k*th iteration, and $t_{p}$ is the true position of the *p*th target.

**Figure 3:**
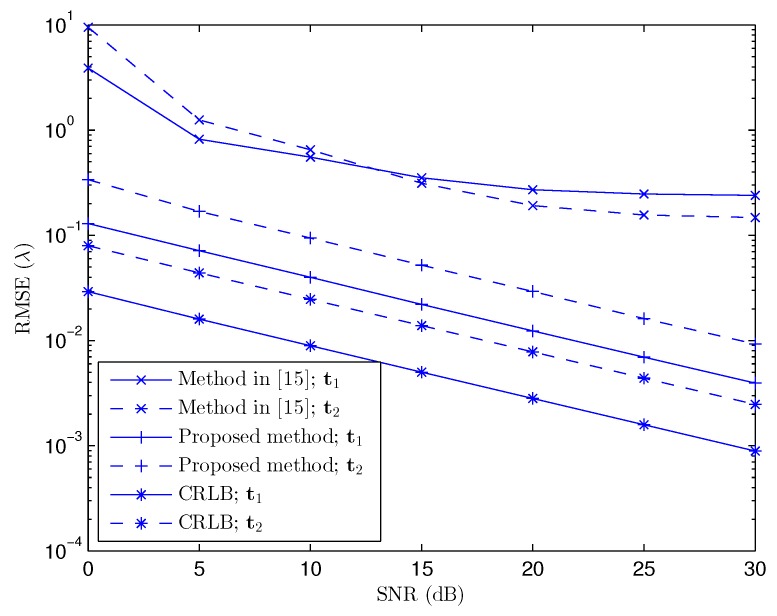
Root mean square error (RMSE) versus signal-to-noise ratio (SNR); $d_{e}=d_{r}=\lambda /4$, K=1000, L=100, M=5, N=9, and P=2. CRLB: Cramér–Rao lower bound.

In Figure 3, we have compared the performance of the proposed method with the method proposed in [15] with two targets at ${\left[\lambda ,\;\lambda ,\;\lambda \right]}^{T}$ and ${\left[2\lambda ,\;1.75\lambda ,\;1.5\lambda \right]}^{T}$ in the Fresnel region. The cost Function (29) has also been applied to [15] to obtain the coordinates. In addition, we have also drawn the Cramér–Rao lower bound (CRLB) in Figure 3, which can be obtained from the existing works [4,13,27]. To keep mathematical analogy with RMSE, we combine the CRLB of the three coordinates of the *p*th target as

(31) $$\begin{array}{c} {\mathrm{CRLB}}_{p}=\sqrt{\sigma_{x_{p}}^{2}+\sigma_{y_{p}}^{2}+\sigma_{z_{p}}^{2}} \end{array}$$

where $\sigma_{x_{p}}^{2}$, $\sigma_{y_{p}}^{2}$, and $\sigma_{z_{p}}^{2}$ denote the CRLB of the corresponding coordinates belonging to the *p*th target.

Figure 3 shows that the proposed method has higher precision and much better performance in terms of RMSE than that of the subspace and approximated model-based method in [15]. This performance gain comes principally from the use of the exact near-field received signal model and PARAFAC decomposition in the proposed technique.

**Figure 4:**
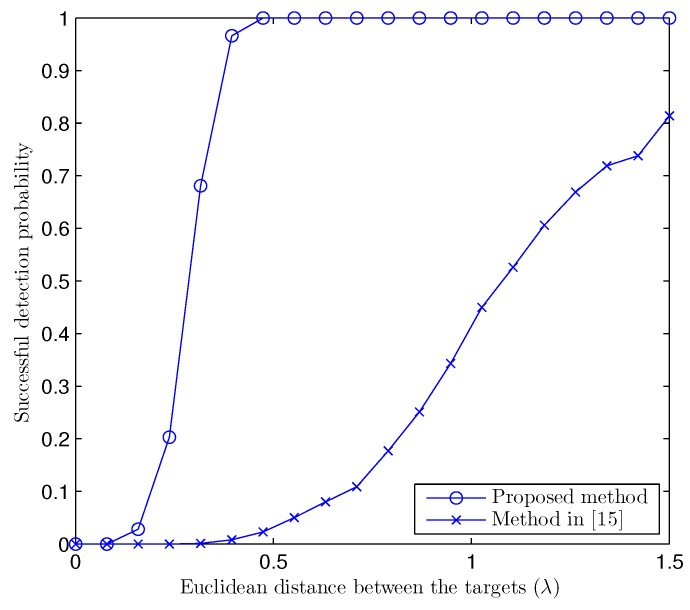
Probability of successful detection versus distance between two targets at SNR =10 dB; $d_{e}=d_{r}=\lambda /4$, K=1000, L=100, M=5, N=9, and P=2.

The resolution capability of a method can be evaluated by the probability of the successful detection P(ξ) of two closely-placed targets, which can be calculated as [12]:

(32) $$\begin{array}{c} P\left(\xi \right)=c\left(\left\{k:{∥{\hat{t}}_{1}\left(k\right)-t_{1}∥}_{F}<\xi \;\mathrm{and}\;{∥{\hat{t}}_{2}\left(k\right)-t_{2}∥}_{F}<\xi \right\}\right)/K \end{array}$$

where k∈{1,2,⋯,K} and $\xi ={∥t_{1}-t_{2}∥}_{F}/2$ is the half of the distance between the two targets. Figure 4 gives the probability of successful detection of two targets at different distances between the targets, which shows that the proposed method has a much better resolution power than its counterpart, even at the high signal-to-noise ratio (SNR) of 10 dB.

## 6. Discussion

In Figure 3, we can observe a significant gap between the RMSE corresponding to the proposed method and the method in [15]. The gain in performance of the proposed method comes from the use of the PARAFAC decomposition and the exact model of the received near-field signals. At high SNR, the method in [15] experiences a floor effect in terms of achievable RMSE performance, which clearly shows the systematic bias introduced by the approximated model. This systematic error is not discernible at low SNR because the major contribution to the estimation error comes from the noise. This bias also explains the low successful detection probability of [15], as shown in Figure 4. Because of the approximation, the location estimated by an approximated model-based method is shifted from the true location, which makes P(ξ) small.

## 7. Conclusions

In this paper, we propose a novel technique for near-field sources localization with a bistatic MIMO system. The principal originalities of this work are the use of the exact model and PARAFAC decomposition for near-field sources localization. Thanks to the exact model of near-field sources, the proposed method has high precision and resolution. The performance of the proposed method greatly surpasses the high-resolution subspace-based method proposed in [15], which proves the importance of an exact model-based method. The proposed method also has some additional advantages with respect to the compared approximated model-based method: it works for the inter-element spacing of λ/2 without any ambiguity, *M* and *N* are not required to be odd, and any antenna can be used as the reference point.

## Abbreviations

The following abbreviations are used in this manuscript:

CRLB	Cramér–Rao Lower Bound
ESPRIT	Estimation of Signal Parameter via Rotational Invariance Techniques
MIMO	Multiple Input Multiple Output
MUSIC	Multiple Signal Classification
PARAFAC	Parallel Factor
RMSE	Root Mean Square Error
SNR	Signal-to-Noise Ratio
TALS	Trilinear Alternating Least Squares
ULA	Uniform Linear Array
